# Inhibitory Effects of KP-A159, a Thiazolopyridine Derivative, on Osteoclast Differentiation, Function, and Inflammatory Bone Loss via Suppression of RANKL-Induced MAP Kinase Signaling Pathway

**DOI:** 10.1371/journal.pone.0142201

**Published:** 2015-11-04

**Authors:** Hye Jung Ihn, Doohyun Lee, Taeho Lee, Sang-Hyun Kim, Hong-In Shin, Yong Chul Bae, Jung Min Hong, Eui Kyun Park

**Affiliations:** 1 Department of Pharmacology, School of Medicine, Kyungpook National University, Daegu, Republic of Korea; 2 College of Pharmacy, Research Institute of Pharmaceutical Sciences, Kyungpook National University, Daegu, Republic of Korea; 3 Department of Oral Pathology and Regenerative Medicine, School of Dentistry, IHBR, Kyungpook National University, Daegu, Republic of Korea; 4 Department of Oral Anatomy and Neurobiology, School of Dentistry, Kyungpook National University, Daegu, Republic of Korea; 5 Skeletal Diseases Genome Research Centre, Kyungpook National University Hospital, Daegu, Republic of Korea; University of Muenster, GERMANY

## Abstract

Abnormally elevated formation and activation of osteoclasts are primary causes for a majority of skeletal diseases. In this study, we found that KP-A159, a newly synthesized thiazolopyridine derivative, inhibited osteoclast differentiation and function *in vitro*, and inflammatory bone loss *in vivo*. KP-A159 did not cause a cytotoxic response in bone marrow macrophages (BMMs), but significantly inhibited the formation of multinucleated tartrate-resistant acid phosphatase (TRAP)-positive osteoclasts induced by macrophage colony-stimulating factor (M-CSF) and receptor activator of nuclear factor-κB ligand (RANKL). KP-A159 also dramatically inhibited the expression of marker genes related to osteoclast differentiation, including TRAP (*Acp5*), cathepsin K (*Ctsk*), dendritic cell-specific transmembrane protein (*Dcstamp*), matrix metallopeptidase 9 (*Mmp9*), and nuclear factor of activated T-cells, cytoplasmic 1 (*Nfatc1*). Moreover, actin ring and resorption pit formation were inhibited by KP-A159. Analysis of the signaling pathway involved showed that KP-A159 inhibited RANKL-induced activation of extracellular signal-regulated kinase (ERK), c-Jun N-terminal kinase (JNK), and mitogen-activated protein kinase kinase1/2 (MEK1/2). In a mouse inflammatory bone loss model, KP-A159 significantly rescued lipopolysaccharide (LPS)-induced bone loss by suppressing osteoclast numbers. Therefore, KP-A159 targets osteoclasts, and may be a potential candidate compound for prevention and/or treatment of inflammatory bone loss.

## Introduction

Osteoclasts are a specialized type of cells capable of resorbing bone, and arise from progenitors of the monocyte/macrophage lineage. Osteoclasts have particular morphological characteristics, such as multiple nuclei, actin rings, and ruffled borders [1,2]. The survival, proliferation, and differentiation of osteoclasts are promoted by two growth factors, M-CSF and RANKL, and the differentiation of BMMs into osteoclasts is primarily governed by the interaction between RANKL and its receptor RANK [3]. RANKL binding to RANK leads to activation of mitogen-activated protein kinases (MAPKs), such as p38, ERK, and JNK; these in turn activate several transcription factors, including nuclear factor kappa-light-chain-enhancer of activated B cells (NF-κB), activator protein-1 (AP-1), and NFAT*c*1, which induce the expression of osteoclast-specific genes [4,5].

Abnormally elevated formation and resorbing activity of osteoclasts induce disruption of the equilibrium between bone formation and resorption. Since the elaborate functions of both bone-forming osteoblasts and bone-resorbing osteoclasts play key roles in bone remodeling for the retention of bone density and quality, enhanced function of osteoclasts can give rise to various skeletal diseases, including osteoporosis, rheumatoid arthritis, and Paget’s disease [1,6,7]. Therefore, targeting or managing the enhanced formation and function of osteoclasts might be effective ways to prevent and remedy skeletal diseases.

Thiazole, a heterocyclic compound that contains both a nitrogen and a sulfur atom, is present in a large variety of natural and synthetic products, such as vitamin B1 and epothilone [8]. Thiazole derivatives are known to exhibit various biological and pharmacological functions, including antifungal, antibacterial, antitubercular, and anti-inflammatory effects [9,10]. Owing to their beneficial effects, various thiazolopyridine derivatives have been developed to deal with a wide range of diseases [11,12]. Thiazolopyridine compounds are able to act as antitumor agents and potential therapeutic agents for Parkinson’s disease, through inhibition of the epidermal growth factor receptor (EGFR) and monoamine oxidase B, respectively [13,14]. In addition, Ohno et al. reported that Ki20227, a compound containing the thiazole ring, suppresses osteoclastogenesis and bone resorption by inhibiting the M-CSF receptor [15].

Therefore, we investigated the effect of KP-A159, a thiazolopyridine derivative, on RANKL-mediated osteoclast differentiation and bone-resorbing activity, and examined the underlying molecular mechanism. In addition, the efficacy of KP-A159 in suppressing inflammatory bone loss was evaluated in mice.

## Materials and Methods

### Reagents and antibodies

**Fig 1:**
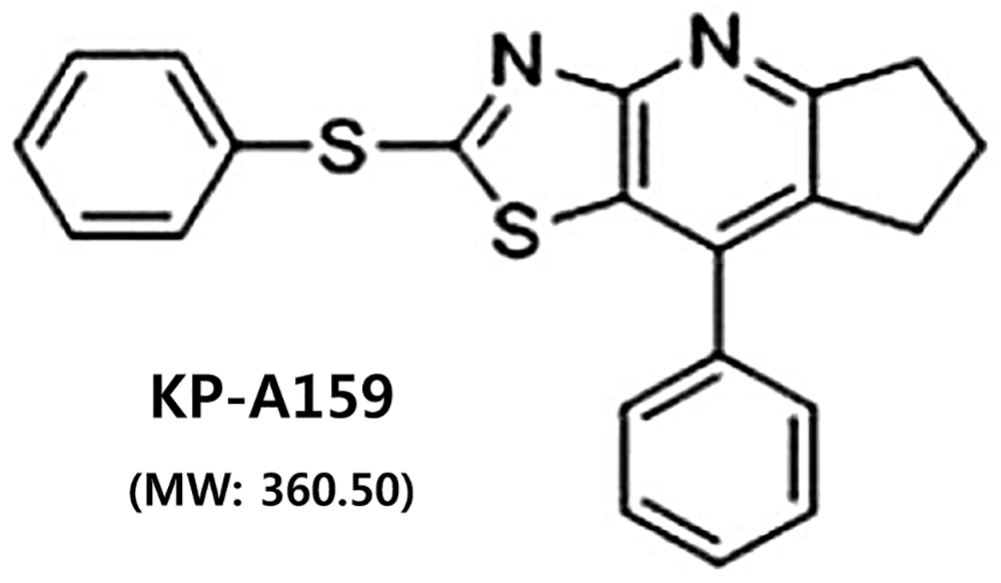
Chemical structure of KP-A159.

Specific antibodies against phospho-p38 (#9211), phospho-JNK (#9251), phospho-ERK (#9106), phospho-MEK1/2 (#9154), and ERK (#9102) were obtained from Cell Signaling Technology (Danvers, MA). Monoclonal anti-β-actin (A5441) was obtained from Sigma—Aldrich (St. Louis, MO). Recombinant mouse M-CSF and RANKL were purchased from R&D Systems (Minneapolis, MN). KP-A159, 8-phenyl-2-(phenylthio)-6,7-dihydro-5*H*-cyclopenta[b]thiazolo[5,4-e]pyridine (Fig 1), is the compound from an in-house chemical library and was synthesized as previously described [16].

### *In vitro* osteoclast differentiation

Osteoclast differentiation was induced as previously described [17]. Bone marrow cells collected from 6–8 week-old C57B6/L mice (Dae Han Bio Link, Chungbuk, Korea) were cultured in α-minimal essential medium (α-MEM) supplemented with 10% fetal bovine serum (FBS). Next day, non-adherent cells were collected, centrifuged in Histopaque density gradient (Sigma—Aldrich, St. Louis, MO), and incubated in α-MEM containing 10% FBS and M-CSF (30 ng/mL) for 3 days. Attached cells were considered to be BMMs. In order to induce osteoclast differentiation, BMMs were cultured in α-MEM supplemented with 20 ng/mL RANKL and 10 ng/mL M-CSF in the absence or presence of 1 μM or 5 μM KP-A159. Osteoclast formation was investigated by TRAP staining following the manufacturer’s instructions (Sigma—Aldrich). TRAP-positive multinucleated cells (MNCs) containing ≥3 nuclei were calculated as osteoclast-like cells.

### Cell viability assay

Cell viability was determined using the methyl-thiazol tetrazolium (MTT) cytotoxicity assay (Sigma—Aldrich). BMMs were incubated with M-CSF (10 ng/mL) either with or without RANKL (20 ng/ml) in the presence or absence of 1 μM or 5 μM KP-A159. After 3 days, MTT was added to each well, the insoluble formazan formed was extracted with dimethyl sulfoxide (DMSO), and absorbance at 570 nm was determined using a 96-well microplate reader (BioRad, Hercules, CA).

### Analyses of gene expression

Total RNA was prepared using TRI-solution (Bioscience, Seoul, Korea) and cDNA was synthesized from 1 μg of total RNA using SuperScript II Reverse Transcriptase (Invitrogen, Carlsbad, CA). Real-time PCR was performed in a LightCycler 1.5 Real-time PCR system (Roche Diagnostics, Rotkreuz, Switzerland) using TOPreal qPCR 2× PreMIX with SYBR green (Enzynomics, Daejeon, Korea). The amplification conditions were as follows: initial denaturation at 95°C for 10 min, followed by 40 cycles of 10 sec at 95°C, 15 sec at 60°C, and 10 sec at 72°C. The primers used for PCR were as previously described [18].

### Western blotting

Cell lysates were prepared using RIPA buffer (10 mM Tris, pH 7.4, 150 mM NaCl, 1% NP-40, 1 mM EDTA, 10% glycerol) containing protease and phosphatase inhibitor cocktail. The lysates (25 μg of protein) were subjected to 10% SDS—PAGE and transfer to nitrocellulose membranes (Whatman, Florham Park, NJ). The membranes were blocked with 3% non-fat milk in TTBS (0.1% Tween 20 in Tris-buffered saline) for 1 h, and then incubated with primary antibodies (1:1000) at 4°C overnight and appropriate secondary antibodies (1:3000) for 1 h. Specific protein bands were detected using WesternBright ECL (Advansta, Menlo Park, CA).

### Staining of actin rings

BMMs placed on glass coverslips were incubated with M-CSF (10 ng/mL) and RANKL (20 ng/mL) with or without 5 μM KP-A159 for 4 days. Cells were then fixed with 4% paraformaldehyde and permeabilized with 0.1% Triton X-100. Actin rings and nuclei were visualized by staining with rhodamine-conjugated phalloidin (Cytoskeleton, Denver, CO) and 4′,6-diamidino-2-phenylindole dihydrochloride (DAPI; Santa Cruz Biotechnology, Santa Cruz, CA), respectively. Images were taken under a BX51 fluorescent microscope (Olympus, Tokyo, Japan).

### Resorption pit assay

BMMs were placed on bone slices (IDS Nordic, Herlev, Denmark) and cultured with M-CSF (10 ng/mL) and RANKL (20 ng/mL) to generate multinucleated osteoclasts. After osteoclasts had formed, cells were treated with or without 5 μM KP-A159 for 2 days. Adherent cells were then eliminated with 1N NaOH for 20 min, and resorption pits were visualized by staining with hematoxylin. The pit area was analyzed using the i-Solution image analysis software (IMT i-Solution, Daejeon, Korea).

### LPS-induced bone loss model and histomorphometric analysis

Animal experiments were performed in accordance to the principles and procedures approved by Kyungpook National University. In order to examine the efficacy of KP-A159 *in vivo*, C57B6/L mice (8 weeks old) were divided into three groups, each consisting of four mice, and treated with the following: the PBS with vehicle, LPS with vehicle, and LPS with KP-A159. LPS (5 mg/kg) was intraperitoneally injected on days 2 and 6. KP-A159 (30 mg/kg) or vehicle was intraperitoneally administered to mice daily for 9 days starting on day 1. At the end of the treatment, all mice were euthanized by pentobarbital overdose, and the right and left femurs of each mouse were excised and fixed in 4% paraformaldehyde for 16 h. The fixed femurs were scanned using a SkyScan 1272 high-resolution micro-computed tomography (μCT) system (Bruker, Kontich, Belgium) with a source voltage of 60kV, current of 166 μA, and resolution of 14 μm. The bone parameters were analyzed using the CTAn software (Bruker, Kontich, Belgium). For histological analysis, the fixed femurs were decalcified and embedded in paraffin, and histological sections (7 μm thickness) were stained with hematoxylin and eosin (H&E) and with TRAP.

### Statistical analyses

Experiments were performed three times and data are presented as the mean ± standard deviation (SD). Statistical analyses were evaluated by the two-tailed Student’s *t*-test or one-way analysis of variance (ANOVA) with Tukey’s multiple comparison post-hoc test. Values *p <* 0.05 or *p <* 0.01 was considered statistically significant.

## Results

### KP-A159 suppresses RANKL-induced osteoclastogenesis

**Fig 2:**
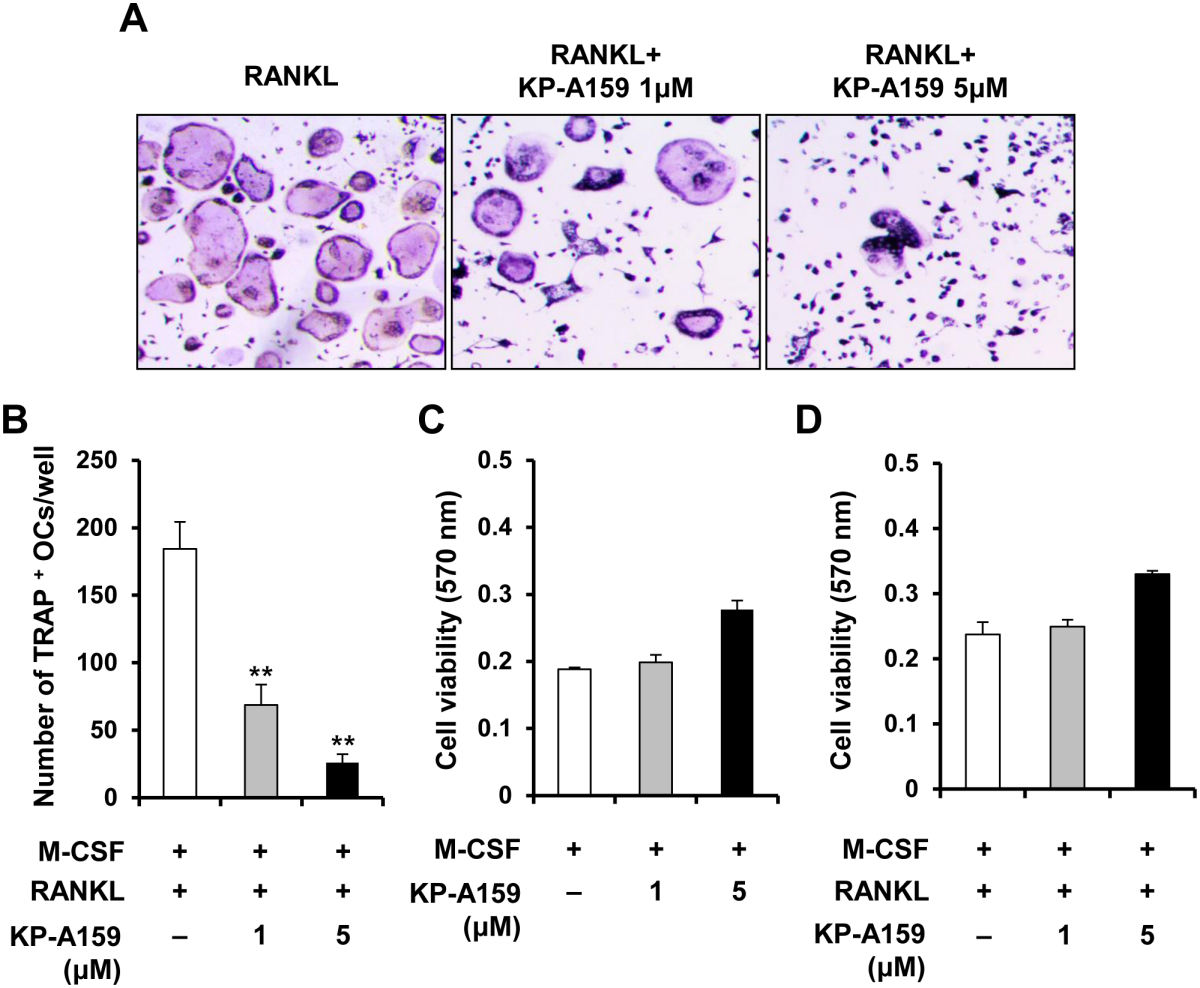
Effects of KP-A159 on RANKL-induced osteoclast differentiation. (A) BMMs were cultured for 4 or 5 days in the presence of M-CSF (10 ng/mL) and RANKL (20 ng/mL) with or without 1 μM or 5 μM KP-A159. Osteoclasts were stained with TRAP. (B) TRAP-positive multinucleated cells with ≥3 nuclei were counted. ** *p* < 0.01 versus vehicle-treated control. (C) and (D) BMMs were cultured for 3 days with M-CSF (10 ng/mL) either with or without RANKL (20 ng/ml) in the presence or absence of 1 μM or 5 μM KP-A159. Cell viability was evaluated by the MTT assay.

To examine the effect of KP-A159 on osteoclast differentiation, we treated BMMs, stimulated with M-CSF and RANKL, with KP-A159 (1 μM or 5 μM) and analyzed the formation of osteoclast-like cells (TRAP-positive MNCs). After 4 days of culture, TRAP-positive MNCs were generated in the positive control (Fig 2A). Compared to the control, the formation of MNCs was considerably reduced by treatment with KP-A159 in a dose-dependent manner, with the number of MNCs being decreased by 62.7% at 1 μM and 85.9% at 5 μM KP-A159 (*p* < 0.01; Fig 2B). The inhibitory effect was not attributable to the cytotoxicity of KP-A159 because the MTT assay showed that KP-A159 (≤5 μM) did not elicit cytotoxic responses in macrophages and pre-osteoclasts (Fig 2C and 2D). These results indicate that KP-A159 dramatically suppresses the generation of osteoclast-like MNCs from BMMs without any cytotoxic effect.

### KP-A159 down-regulates the expression of osteoclast marker genes, and suppresses formation of actin rings

**Fig 3:**
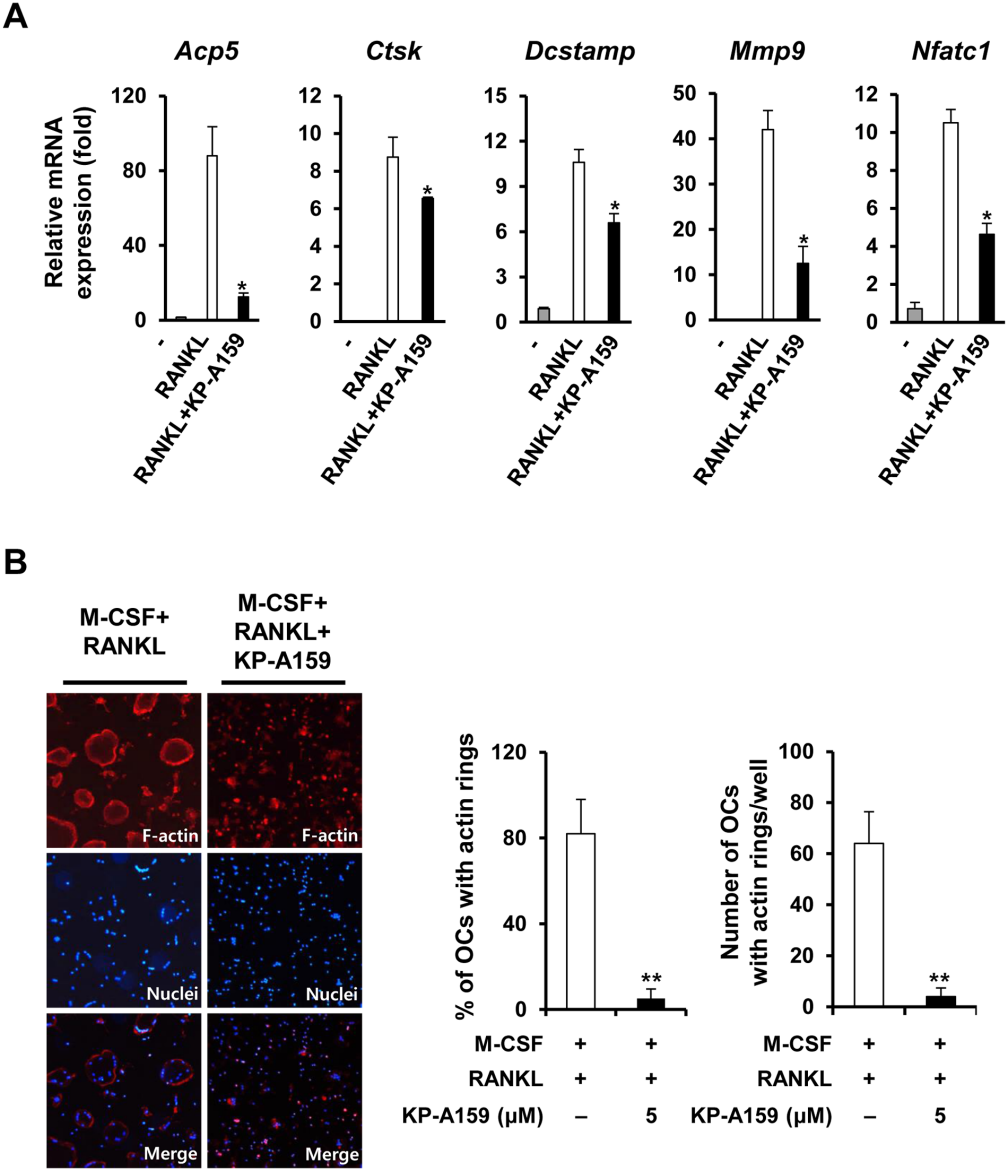
Effects of KP-A159 on the mRNA expression of osteoclast marker genes, and on the formation of actin rings. (A) The mRNA expression of TRAP (*Acp5*), Cathepsin K (*Ctsk*), DC-STAMP (*Dcstamp*), MMP9 (*Mmp9*), and NFATc1 (*Nfatc1*) was analyzed using real-time RT-PCR. * p < 0.05. (B) BMMs seeded on glass coverslips were incubated for 4 days with M-CSF (10 ng/mL) and RANKL (20 ng/mL) in the presence or absence of KP-A159 (5 μM). The cells were stained with rhodamine-conjugated phalloidin and DAPI to identify actin rings and nuclei, respectively. ** *p* < 0.01 versus vehicle-treated control.

We examined effect of KP-A159 on the expression of various genes that are related to osteoclast differentiation. Expression of the mRNAs encoding osteoclast-specific markers, such as TRAP (*Acp5*), Cathepsin K (*Ctsk*), DC-STAMP (*Dcstamp*), MMP9 (*Mmp9*) and NFATc1 (*Nfatc1*) was markedly increased during RANKL-induced osteoclast differentiation (Fig 3A). However, the addition of KP-A159 significantly suppressed RANKL-induced mRNA expression of these genes compared to the positive control, indicating that KP-A159 effectively inhibits osteoclast differentiation through the down-regulation of the expression of osteoclast marker genes (Fig 3A). Since DC-STAMP, which is vital for the fusion of pre-osteoclasts, was down-regulated by KP-A159, we also tested the effect of KP-A159 on formation of multinucleated giant cells and cytoskeletal reorganization. The cells treated with KP-A159 hardly formed MNCs as well as actin rings (Fig 3B). Osteoclasts containing actin ring were decreased by 93% (Fig 3B). These results suggest that KP-A159 inhibits osteoclast differentiation.

### KP-A159 attenuates bone resorption activity

**Fig 4:**
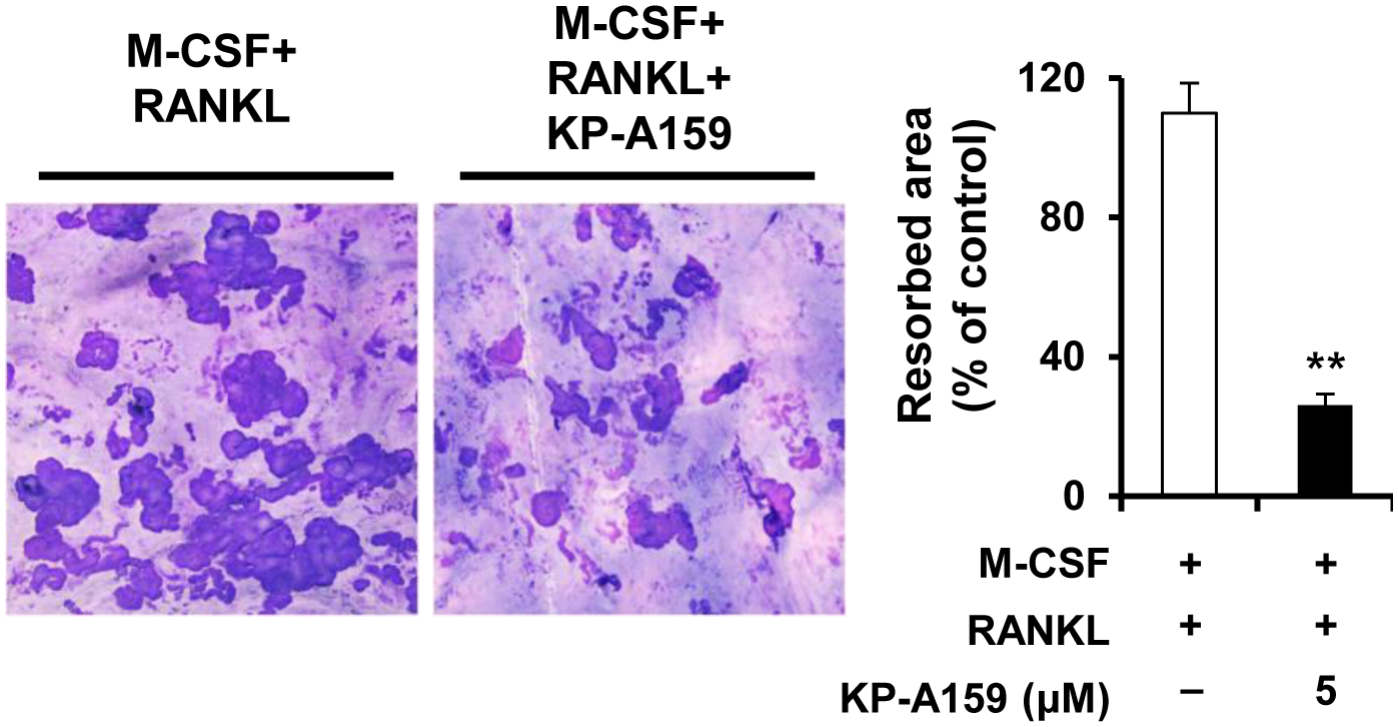
Effects of KP-A159 on the formation of resorption pits. BMMs were plated onto bone slices and incubated with M-CSF (10 ng/mL) and RANKL (20 ng/mL) for 3 days to induce differentiation into osteoclasts. The cells were treated with or without KP-A159 (5 μM) for an additional 2 days. Resorption pits were visualized by staining with hematoxylin. ** *p* < 0.01 versus vehicle-treated control.

Once mature osteoclasts are formed, they are capable of resorbing mineralized tissue. To determine if KP-A159 affects the resorbing activity of osteoclasts, we carried out the resorption pit assay. BMMs were incubated on bone slices in osteoclast-inducing medium for 3 days to generate osteoclast-like MNCs, and then treated with KP-A159 or vehicle for an additional 2 days. As shown in Fig 4, KP-A159 treatment markedly inhibited the formation of resorption pits compared to the positive control (76.2% reduction). The result suggests that KP-A159 strongly suppresses the bone-resorbing activity of osteoclasts.

### KP-A159 inhibits RANKL-stimulated phosphorylation of MEK, ERK and JNK

**Fig 5:**
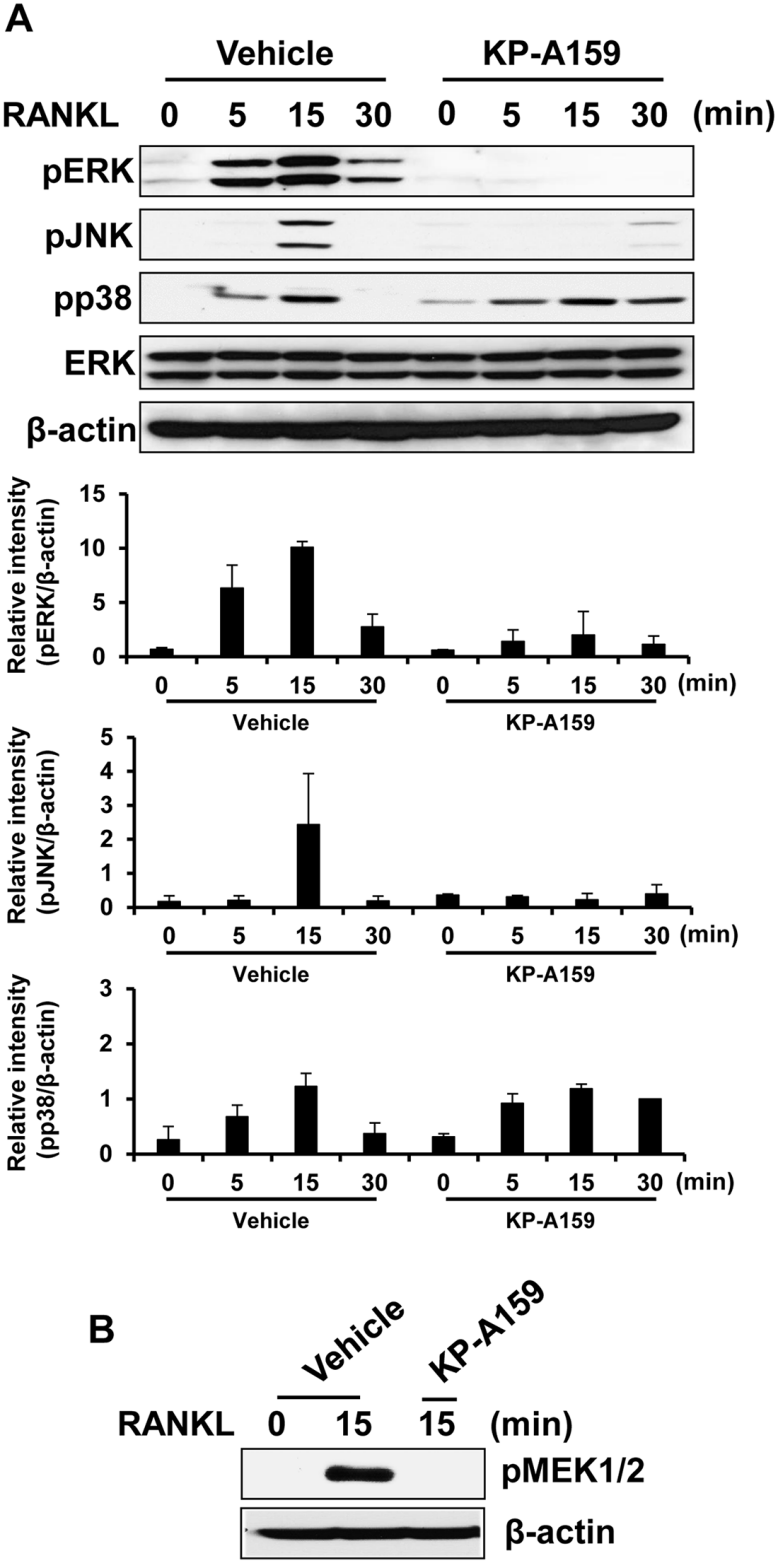
Effects of KP-A159 on RANKL-induced signaling. (A) and (B) Murine BMMs were incubated in serum-free medium for 5 h, then pretreated with KP-A021 (5 μM) or vehicle for 1 h before RANKL (50 ng/mL) stimulation for the indicated times. Phosphorylation of ERK, JNK, p38, and MEK1/2 was determined by western blot. Total ERK or β-actin was used as the loading control.

The activation of MAPKs, including p38, ERK, and JNK, is required for the induction of osteoclastogenesis. To examine how KP-A159 suppresses RANKL-mediated osteoclast differentiation, the phosphorylation of MAPKs in response to RANKL was analyzed. RANKL induced phosphorylation of p38, ERK, and JNK after 15 min of RANKL stimulation in the positive (vehicle-treated) control (Fig 5A). Pretreatment with KP-A159 decreased RANKL-induced ERK and JNK phosphorylation, whereas phosphorylation of p38 was not down-regulated by pretreatment with KP-A159 (Fig 5A). Phosphorylation of p38 was rather sustained by 30 min. In addition, activation of MEK1/2, upstream signaling molecule of ERK, was also markedly suppressed by KP-A159 (Fig 5B). These results suggest that KP-A159 inhibits activation of the MEK-ERK cascade as well as JNK phosphorylation, leading to suppression of RANKL-induced osteoclast differentiation.

### KP-A159 prevents LPS-induced bone loss *in vivo*

**Fig 6:**
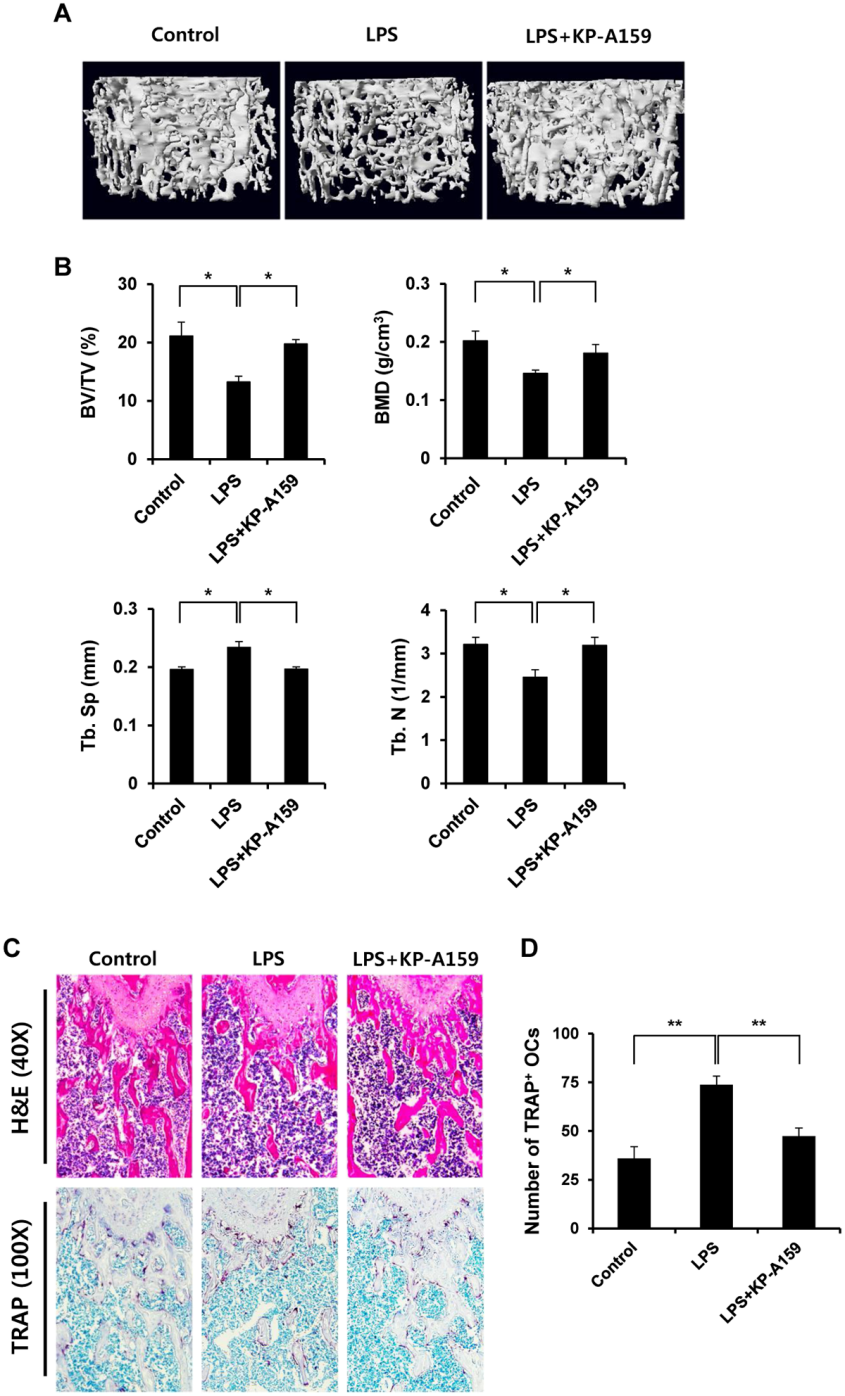
Effect of KP-A159 on LPS-induced bone loss. (A) Mice were sacrificed 8 days after the first LPS injection, and three-dimensional images of femurs were obtained using μCT. (B) Bone volume per tissue volume (BV/TV), bone mineral density (BMD), trabecular separation (Tb. Sp), and trabecular number (Tb. N) were analyzed using the CTAn software. n = 4 (eight legs) in each group. * *p* < 0.05. (C) Fixed femurs were decalcified and sectioned. Sections were stained with H&E (40x) and TRAP (100x). (D) The number of TRAP-positive osteoclasts was assessed. ** *p* < 0.01.

Since KP-A159 treatment suppressed RANKL-induced osteoclastogenesis as well as the resorbing activity of mature osteoclasts *in vitro*, we asked whether the inhibitory effect of KP-A159 would also be exhibited *in vivo*. To determine the efficacy of KP-A159 *in vivo*, LPS-induced bone loss analysis was performed. As shown in Fig 6A, LPS administration apparently caused trabecular bone loss in femurs. On the other hand, KP-A159 alone did not affect the quality of trabecular bone, and LPS-induced bone loss was considerably decreased by co-injection of KP-A159 (S1 Fig and Fig 6A). In correlation with μCT images, the reduction of bone volume per tissue volume (BV/TV), bone mineral density (BMD), and trabecular number (Tb. N) by LPS injection were recovered in KP-A159-treated mice (Fig 6B). Histological sections also showed the suppressive activity of KP-A159 on the trabecular bone loss caused by LPS (Fig 6C). In addition, the increased number of TRAP positive osteoclasts induced by LPS was significantly reduced in KP-A159-treated mice (Fig 6D), suggesting that KP-A159 has an inhibitory effect on osteoclast formation *in vivo*. These results demonstrate that KP-A159 suppresses LPS-induced bone loss *in vivo*, through inhibition of osteoclast formation and bone resorption.

## Discussion

Diverse therapeutic agents, including bisphosphonates and calcitonin have been developed and used to treat bone diseases that are primarily caused by enhanced formation and activation of osteoclasts [19]. Although they have been shown to exhibit beneficial effects, there are limitations to their use due to severe or mild adverse events, including osteonecrosis of the jaw and hypocalcemia [20,21]. Thus, the development of an alternative agent that suppresses osteoclastogenesis as well as the resorbing activity of mature osteoclasts, and has few or no adverse effects, is required. In the present study, we report that the thiazolopyridine derivative, KP-A159, considerably suppresses RANKL-induced differentiation and function of osteoclasts, and its inhibitory effect is mediatied by suppressing the MEK/ERK and JNK pathways.

Critical transcription factors such as AP-1 (Fos-Jun heterodimer), NF-κB, and NFAT*c*1, promote the expression of genes that are strongly implicated in the formation of multinucleated osteoclasts and in resorbing activity. In particular, several studies have proven that NFAT*c*1 acts as the chief transcription factor in osteoclastogenesis. NFAT*c*1 deficiency leads to the failure of the differentiation of embryonic stem cells into osteoclasts in response to RANKL, and *Nfatc1* conditional knockout mice exhibit osteopetrosis [22,23]. In the present study, we found that KP-A159 strongly inhibited the expression of RANKL-stimulated *Nfatc1*, and other osteoclast marker genes, including *Acp5*, *Ctsk*, *Dcstamp*, and *Mmp9* (Fig 3A). DC-STAMP is vital for the cell-cell fusion that generates multinucleated osteoclasts and for bone resorption [24,25]. KP-A159 treatment suppressed the expression of DC-STAMP (Fig 3A), and consequently, disrupted the formation of both multinucleated osteoclasts (Fig 2B) and actin rings (Fig 3B). Our results indicate that KP-A159 might down-regulate the expression of NFAT*c*1, thereby suppressing osteoclastogenesis and impairing cell-cell fusion.

Binding of RANKL to RANK instigates the activation of MAPKs implicated in the transcriptional regulation of genes during osteoclastogenesis [6]. Among MAPK signaling pathways, the ERK pathway is known to be involved in the activation of c-Fos, which is a component of AP-1, and the AP-1 complex elicits the induction of NFAT*c*1 [26–28]. Previous studies have shown that ERK inhibition or its genetic deletion induces decreased osteoclast formation and bone-resorbing activity [27,29,30]. The other element of the AP-1 complex, c-Jun, is activated by the RANKL-stimulated JNK signaling pathway, and Ikeda et al. showed the essential role of c-Jun signaling in osteoclastogenesis *in vivo* and *in vitro* [31]. Thus, these studies indicate that RANKL-mediated ERK and JNK signaling pathways play critical roles in osteoclast differentiation and function. In our results, KP-A159 significantly blocked the phosphorylation of JNK, ERK, and its upstream signaling molecule, MEK (Fig 5). On the other hand, phosphorylation of p38 was not inhibited by KP-A159, and sustained until 30 min after RANKL stimulation. This might be due to increased basal level of phosphorylation by KP-A159 (Fig 5). Although phosphorylation of p38 was not inhibited by KP-A159, the MEK-ERK cascade and JNK phosphorylation were dramatically inhibited by KP-A159, and this might be major events contributing to the inhibition of both osteoclastogenesis and the resorbing activity of mature osteoclasts by KP-A159.

Recently, Hamade et al. reported that CX-32 and CX-35, thiazole derivatives which are structurally distinct from KP-A159, exhibit anti-inflammatory effects by inhibiting prostaglandin production in LPS-stimulated RAW 264.7 macrophages [32]. In certain pathological cases, inflammation causes bone erosion. In a mouse model, LPS is known to be a potent inducer of bone destruction *in vivo*, and its stimulus elicits the inflammatory response by promoting the production of inflammatory mediators. As expected, KP-A159 attenuated LPS-mediated bone destruction *in vivo*, whereas KP-A159 alone has nearly no effect on the quality of trabecular bone (Fig 6A and S1 Fig). Reduction of bone destruction was accompanied with reduced formation of osteoclast (Fig 6C and 6D), suggesting that KP-A159 also suppresses LPS-induced osteoclast formation *in vivo*. Taken together, our results demonstrate that KP-A159 could serve as a beneficial agent for reducing inflammatory bone destruction.

In summary, we have demonstrated that KP-A159, a thiazolopyridine derivative, inhibits osteoclast differentiation of BMMs by blocking activation of the MEK-ERK cascade and the JNK signaling pathway induced by RANKL. KP-A159 also disrupted actin ring formation, and reduced bone resorption of osteoclasts. Furthermore, KP-A159 attenuated LPS-induced bone destruction *in vivo*. Our findings suggest that KP-A159 may be a promising pharmacological agent for the treatment of bone diseases that are related to elevated osteoclast formation and resorption.

## Supporting Information

S1 Fig Effect of KP-A159 on trabecular bone quality. (A) Control or KP-A159-treated mice were sacrificed, and three-dimensional images of femurs were obtained using μCT. (B) Bone volume per tissue volume (BV/TV), bone mineral density (BMD), trabecular separation (Tb. Sp), and trabecular number (Tb. N) were analyzed using the CTAn software. n = 4 (eight legs) in each group.
(TIF)
