# Supplementary figures and images for: Inhibitory Effects of KP-A159, a Thiazolopyridine Derivative, on Osteoclast Differentiation, Function, and Inflammatory Bone Loss via Suppression of RANKL-Induced MAP Kinase Signaling Pathway
Source: PLoS One. 2015 Nov 4;10(11):e0142201. doi: 10.1371/journal.pone.0142201 (PMC4633183; doi:10.1371/journal.pone.0142201)

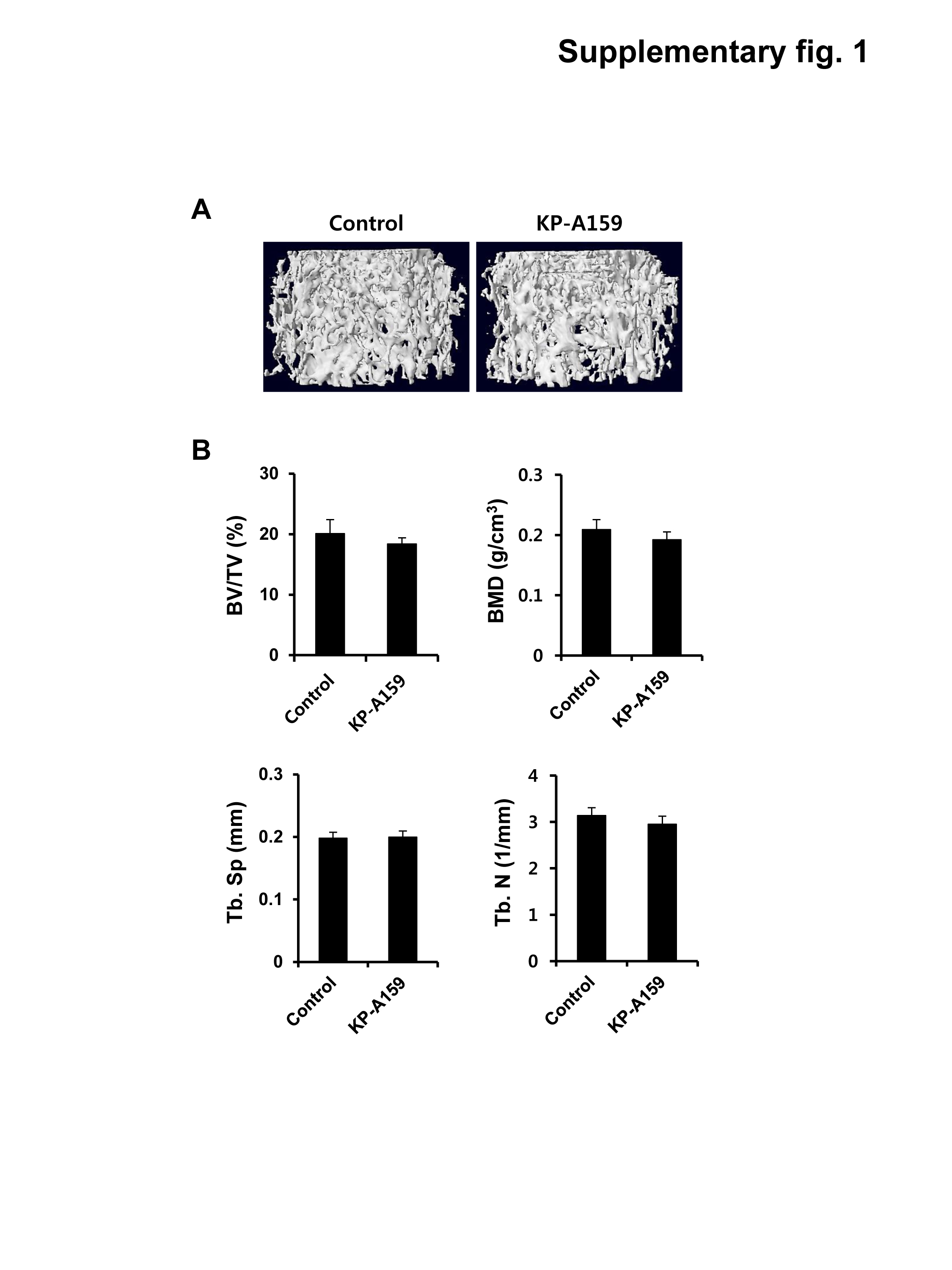

Supplement: S1 Fig — (A) Control or KP-A159-treated mice were sacrificed, and three-dimensional images of femurs were obtained using μCT. (B) Bone volume per tissue volume (BV/TV), bone mineral density (BMD), trabecular separation (Tb. Sp), and trabecular number (Tb. N) were analyzed using the CTAn software. n = 4 (eight legs) in each group. (TIF) [file pone.0142201.s001.tif]
